# Feasibility of Establishing HIV Case-Based Surveillance to Measure Progress Along the Health Sector Cascade: Situational Assessments in Tanzania, South Africa, and Kenya

**DOI:** 10.2196/publichealth.7610

**Published:** 2017-07-10

**Authors:** Richelle Harklerode, Sandra Schwarcz, James Hargreaves, Andrew Boulle, Jim Todd, Serge Xueref, Brian Rice

**Affiliations:** ^1^ University of California San Francisco Global Health Sciences San Francisco, CA United States; ^2^ San Francisco Department of Public Health San Francisco, CA United States; ^3^ London School of Hygiene and Tropical Medicine London United Kingdom; ^4^ University of Cape Town Cape Town South Africa; ^5^ World Health Organization Geneva Switzerland

**Keywords:** HIV, surveillance, case reports, continuum of care, epidemiologic surveillance

## Abstract

**Background:**

To track the HIV epidemic and responses to it, the World Health Organization recommends 10 global indicators to collect information along the HIV care cascade. Patient diagnosis and medical record data, harnessed through case-based surveillance (CBS), can be used to measure 8 of these. While many high burden countries have well-established systems for monitoring patients on HIV treatment, few have formally adopted CBS.

**Objective:**

In response to the need for improved strategic HIV information and to facilitate the development of CBS in resource-limited countries, we aimed to conduct situational assessments of existing data collection systems in Tanzania, South Africa, and Kenya.

**Methods:**

We developed a standardized protocol and a modularized data collection tool to be adapted for the particular focus of the assessments within each country. The three countries were selected based on their stage of readiness for CBS. The assessment included three parts: a desk review of relevant materials on HIV surveillance and program monitoring, stakeholder meetings, and site visits.

**Results:**

In all three countries, routine HIV program monitoring is conducted, and information on new HIV diagnoses and persons accessing HIV care and treatment services is collected. Key findings from the assessments included substantial stakeholder support for the development of CBS, significant challenges in linking data within and between systems, data quality, the ability to obtain data from multiple sources, and information technology infrastructure. Viral load testing capacity varied by country, and vital registry data were not routinely linked to health systems to update medical records.

**Conclusions:**

Our findings support the development of CBS systems to systematically capture routinely collected health data to measure and monitor HIV epidemics and guide responses. Although there were wide variations in the systems examined, some of the current program and patient monitoring systems can be adapted to function effectively for CBS, especially if supported by an improved patient registration system with shared unique health identifiers.

## Introduction

The World Health Organization (WHO) released consolidated guidelines in 2015 recommending ten global indicators to track the HIV epidemic and responses to it and measure progress and drive action toward the 90-90-90 targets of the United Nations Joint Program on AIDS (UNAIDS) [1,2]. To provide high-quality, timely, and reliable data by population characteristics and across the different levels of a health care system, it is recommended to develop a comprehensive strategic HIV information system [2-7]. Case-based surveillance (CBS) is such a system, and it is a key element of HIV Second Generation Surveillance, which is recommended by the WHO [8].

Case-based surveillance has three characteristics that differentiate it from most HIV program monitoring systems. The main distinctive characteristic is that at all levels (facility, subnational, and national health agencies or ministries), CBS systems obtain and retain *individual-level data* for each person diagnosed with HIV. The second characteristic is *within-system record linkage* that is facilitated by the collection of a unique identification number (eg, a national or regional health care identification number) or unique personal identifiers (eg, full or part date of birth, sex, full or part name, and a marker of residence). Individual record linkage at the national level enables the identification and removal of duplicate records and the tracking of cases over time, thereby providing a more accurate number of cases than can be obtained through aggregate data from facilities. The third characteristic is that CBS data are gathered and retained from *multiple data sources* (eg, from testing laboratories and facilities and from care facilities). This aspect of CBS serves two important purposes: it improves the completeness of case ascertainment, and it provides critical information on the key events that concur with global indicators [1]. These events include HIV diagnosis, entry into care, initiation of antiretroviral therapy (ART), disease progression or treatment success (as measured by CD4 (T-helper) cell and viral load tests), and death.

By developing a linked database, CBS data can contribute information to all but two (*domestic finance and prevention by key population*) of the ten global indicators described in the consolidated guidelines [1]. Table 1 presents how CBS data may be utilized to measure the eight indicators [1].

**Table 1:** The use of case-based surveillance data in measuring indicators along the HIV care cascade.

Indicator	CBS^a^ data
People living with HIV	Directly provide national or regional specific estimates of people living with diagnosed HIV and indirectly provide national or regional specific estimates of undiagnosed HIV by fitting statistical models to data (eg, back-calculation analysis of CD4 count at the time of diagnosis)
Knowing HIV status	Indirectly provide estimate of denominator (all living with HIV) and directly provide estimate of numerator (cumulative diagnoses minus deaths or number of people currently in care)
Linkage to care	Directly measured based on the report of a CD4 cell or viral load test
Currently on ART^b^	Indirectly provide estimate of denominator (all living with HIV) and directly provide estimate of numerator (number of people currently on ART)
ART retention	Directly measure denominator (initiated ART) and numerator (number of people retained on ART according to an applied-time criteria)
Viral suppression	Directly calculate denominator (on ART) and numerator (virally suppressed)
AIDS deaths	Directly measure through reporting (the provision of data from source) and follow-up of cases, and indirectly measure through linkage with vital statistics for deaths and cause of death
New HIV infections	Provide a framework for conducting recency testing or for incidence estimation through back-calculation analysis of CD4 count at the time of diagnosis

^a^CBS: case-based surveillance.

^b^ART: antiretroviral therapy.

Although HIV CBS is conducted in many high, middle, and low income countries, it has not been implemented in sub-Saharan Africa, where the burden of the disease has been greatest [9]. In an environment where donors are increasingly insisting on cost-sharing assurances from the governments of low- and middle-income countries, and for more cost-effective use of funds [1], HIV CBS presents a cost-effective method for collecting strategic information in sub-Saharan Africa to improve patient and program management. To identify systems that are context appropriate, feasible, scalable, and sustainable for CBS, the Measurement and Surveillance of HIV Epidemics (MeSH) Consortium facilitated situational assessments in Tanzania, South Africa, and Kenya between August 2015 and February 2016.

## Methods

In our assessments, we focused on the feasibility of implementing CBS, including the availability of individual-level data, the ability to uniquely identify and link cases, and the capacity to capture key events along the HIV care cascade from multiple sources. We developed a protocol and data collection tool to promote a standardized approach to our situational assessments [10]. Our assessment included three parts: (1) desk review of materials relevant for HIV surveillance, (2) meetings with stakeholders knowledgeable about HIV strategic information, (3) site visits to understand human resource capacity and the availability and quality of data. For the meetings and site visits, the data collection tool was used and notes were taken by between two and four assessors while on-site. In Kenya and South Africa, all meetings were conducted in English. In Tanzania, the majority of the meetings where conducted in English, with a couple conducted in Kiswahili with the aid of a translator.

The data collection systems assessed include patient monitoring systems (PMS), which maintain individual patient health information at the facility, and laboratory information management systems (LIMS), which maintain individual-level data from laboratory tests. Information held by PMS may be paper-based (consisting of a standard medical chart) or electronic—referred to as an electronic medical record (EMR)—and may be completed either by clinical or clerical staff after clinical staff complete the paper records.

Our assessment team had experience in the implementation and management of PMS and CBS systems and data. Prior to each assessment, we met with national and international stakeholders to confirm the focus of the appraisal, to develop a list of documents to obtain and review (eg, on policies concerning disease reporting, medical record privacy, and data security), to identify further relevant stakeholders for engagement, and to agree on sites to visit.

Kenya, Tanzania, and South Africa were selected for assessment based on having high HIV prevalence, partial or fully implemented electronic data systems, and being a MeSH Consortium focus country. The focus of our assessment differed in each country (see below). In all 3 countries, there was engagement with regional and national stakeholders, including Ministry of Health representatives, and results with recommendations were presented back to stakeholders in the form of a country specific detailed assessment report.

In Tanzania, we assessed the feasibility and acceptability of developing a HIV CBS system. As the HIV care and treatment clinic (CTC) PMS has greatest coverage of individual-level data in Tanzania, the system was a major focus of our assessment. To include both urban and rural areas with substantial disease burden, the assessment was conducted in the Dar-es-Salaam and Mwanza regions. Site visits were made to nine health facilities and laboratories providing HIV services in these areas at various levels (regional and district hospitals, health centers, and dispensaries). A total of 23 stakeholders were engaged with at the national, regional, district, and facility levels.

Our assessment in South Africa considered how existing multi-facility-based integrated HIV data systems and various patient identifiers could be used for CBS. We focused on the Three Interlinked Electronic Registers system (TIER.Net) and the National Health Laboratory System (NHLS). The Western Cape Province was selected as a region where the TIER.Net system and identifiers could be explored. The NHLS is based in Johannesburg. Seven stakeholders were engaged at the national and regional (Western Cape) level and two health facilities offering HIV care and treatment and one regional laboratory were visited.

In Kenya, in 2015, a pilot of CBS was performed in a high-burden region in order to inform the development and implementation of a national CBS system. Cases were identified and reported by surveillance officers and by facility staff who received a monetary stipend for their CBS work. Among other findings, the human resource costs associated with the pilot methods called into question the sustainability of this CBS model. The goal of our assessment was to explore reporting options that might be less demanding of human resources. In Nairobi and Kisumu counties, we examined the potential for reporting from EMRs, laboratories, and providers (clinical and HIV testing counselors) without additional compensation and met with six national and regional stakeholders. We also visited three regional laboratories and 8 health facilities at various levels (sub-district hospitals, health centers, and dispensaries).

We present results and recommendations from the three components of our assessments in each country and by a framework based on the three distinctive characteristics of CBS. In addition, we assess the commonalities and differences in the ability of the three countries to measure the care cascade indicators and the feasibility of implementing CBS under current conditions.

## Results

### United Republic of Tanzania

#### Collecting Individual-Level Data

Currently, in Tanzania, the most comprehensive mechanism for reporting clinical HIV data is aggregate reporting of routine program monitoring activities to the District Health Information System (DHIS-2). In addition to this, HIV programs led by the Ministry of Health and Social Welfare (MoHSW) have their own reporting mechanisms. Primary among these is the HIV CTC program, which is the PMS for HIV care and treatment that collects individual-level data for both adult and pediatric cases. At the time of the assessment, 637 CTC sites reported individual-level data to the national CTC system, providing information on 67% of all persons receiving ART [11].

#### Uniquely Identifying and Linking Records

Names are collected when patients enter HIV care, but are not made available outside of a health facility. Names are not universally collected at HIV testing and counseling (HTC) facilities. The recording of names of persons receiving HTC services in a paper registry was observed at one facility, although the purpose of this was not clear. While information on age and sex are collected at testing and care facilities, the absence of names at testing sites presents challenges for linking records to obtain an accurate estimate of the number of people diagnosed or to monitor linkage to care.

Each CTC facility records personal identifiable information and assigns a unique number to new attendees, thereby facilitating within-site data linkage. Although this CTC number is unique within the country, it is often not transferred from one facility to the next, resulting in patients being assigned an additional number when obtaining care at a subsequent CTC site. Multiple assignments of CTC numbers, coupled with the absence of a national identifier in Tanzania, make data linkage between sites to monitor individuals who receive care at two or more sites consecutively or over time, challenging.

#### Capturing Key Events From Multiple Data Sources

A number of variables required for CBS to produce key indicators along the care cascade are available through the CTC. These include the number of patients in care, the number of patients on ART, and ART retention. As identifying data are not routinely collected at HTC facilities, individual-level data are only captured for those who seek care. Tanzania has not yet adopted routine viral load monitoring for patients on ART.

Most CTC facilities enter data into an electronic database. However, various information technology issues were observed, including smaller facilities not having computers, inconsistent connectivity and power outages, and a lack of interoperability between the PMS and other data systems at the health facility. It is likely that the observed shortcomings of the health facility staff members in the area of information technology compromises the completeness and quality of the data. Neither new diagnoses nor vital statistics data are linked with CTC data, and although regional laboratories and pharmacies have electronic information systems, they also are not linked to the CTC system.

#### Feasibility and Readiness of Implementing Case-Based Surveillance

The CTC system currently performs several of the functions of CBS, collecting individual-level data from point of entry into care on approximately two-thirds of persons on ART in Tanzania. To move toward a comprehensive system for CBS, CTC data would need to be routinely and accurately linked across CTC and non-CTC facilities, HTC programs, and vital statistics.

The situational assessment found that CBS implementation would put pressure on resources and staff time and would potentially undermine clinical care and data quality if there were additional data collection requirements. Current data quality was a concern, as data quality assurance (DQA) through availability of, and adherence to, standard operational procedures, is not enforced, and there is limited use of data beyond mandated program reporting.

### Republic of South Africa

#### Collecting Individual-Level Data

South Africa has implemented a national PMS that captures longitudinal information on persons on ART. The system, entitled TIER.Net, collects individual-level data at the facility level that are then reported nationally, including patient names and other personal identifiers. Although TIER.Net has modules for HIV testing and pre-ART care, neither was in operation in the places we assessed. Reportedly, these modules are used by some jurisdictions.

In South Africa, HIV testing is based on a point-of-care rapid-testing algorithm, with only discordant findings sent to a laboratory for confirmation. Health facilities do, however, use a robust and reliable transport system to routinely send specimens to regional laboratories for CD4 cell counts, viral loads, early infant diagnosis, full blood counts, and blood chemistry. As part of the NHLS, the national laboratory data warehouse has available all CD4 cell count and viral load tests from persons receiving HIV care in the public sector. The NHLS provides services to over 80% of the population (and a higher percentage of those living with HIV) through a national network of laboratories that use a LIMS to capture and centralize individual-level data for both adult and pediatric patients.

Other individual-level data sources are available nationally and regionally. In the Western Cape, individual-level data on hospital admissions, ambulatory visits, and medicine dispensing are accessible to government health services with varying completeness. Vital registration systems are well established in South Africa, with 96% of HIV deaths estimated to be recorded in a national population register [12]. These data, however, are no longer routinely available to health services as death certificates are now sealed and managed by other government departments after completion by health practitioners.

#### Uniquely Identifying and Linking Records

There are a number of distinct personal identifiers, HIV-specific and general, in use in South Africa. Health services issue medical record numbers that are unique to each facility. The national civil identifier is available to all citizens and is currently recorded as an identifier when patients register for health services. However, approximately 10% of the South African population are non-citizens and, therefore, are not assigned this identifier. Moreover, a substantial proportion of citizens have historically not presented their civil identification documents when availing themselves of services. A national health patient registration system is currently being developed, in which every patient will be eligible.

Recognizing the limitations of the national civil identifier and facility medical record numbers for resolving the issue of duplication of cases for surveillance purposes, the NHLS data warehouse considers name and date of birth alongside this identifier to distinguish unique individuals. The NHLS also includes barcodes that can be scanned for each test that uniquely identify the patient specimens and assist with resolving duplication issues and establishing the data quality of identifiers.

Patient master indexes are in use in some areas to identify and resolve duplication of records pertaining to a single patient. For example, in the Western Cape Province, a patient master index has enabled linkage of laboratory, pharmacy, and service data in a data center environment that meets the key requirements of CBS.

#### Capturing Key Events From Multiple Data Sources

The variables required by CBS to produce a number of the indicators along the care cascade are available. The PMS can be used to identify the number of patients on ART, ART retention, and viral suppression. Viral load and CD4 testing is standard for ART patients and is performed at centralized laboratories using the same national LIMS. Having been de-duplicated, the NHLS uses CD4 test data to estimate the number of persons in care and viral load test data to estimate the number of persons on ART and to measure viral suppression. The number of persons newly diagnosed with HIV is only captured in registers at health facilities, as diagnosis is done by rapid testing.

#### Feasibility and Readiness of Implementing Case-Based Surveillance

Both the PMS and NHLS LIMS perform several of the functions of CBS, collecting individual-level data and routinely de-duplicating these data at the provincial and national level. To build a CBS system based on these systems, all point-of-care test results (not only discordant test results) will require to be digitalized for entry into the LIMS. To comprehensively describe the care pathway, data from vital statistics programs will also need to be routinely linked to these data. A CBS system built on laboratory and patient information systems would obviate the need for developing a formal notification process for surveillance purposes, an activity that has historically failed in South Africa.

The PMS has staff shortages and facility service pressures that could compromise adherence to standard operating procedures and, therefore, data completeness and quality. There is a backlog of laboratory results to be filed in medical charts in many facilities, resulting in incomplete laboratory data being reported through the PMS.

### Republic of Kenya

#### Collecting Individual-Level Data

Multiple data systems exist in Kenya from which individual-level data for HIV CBS can be obtained. Information on new HIV diagnoses for both adult and pediatric cases is captured in paper registers at health facilities. Individual-level HIV care and treatment data are collected at facilities using standard Ministry of Health forms and registers, although the data are reported in aggregate at the national level. In the majority of the large HIV care facilities (>500 patients), EMRs have been implemented. Facilities with EMRs report quarterly to a data warehouse that was developed in 2015. At the time of the assessment, 347 (52%) of the EMR facilities were reporting data to the warehouse. There are also seven regional laboratories that conduct viral load and early infant diagnostic tests, all using a LIMS to capture patient-level data.

Existing infrastructure presents challenges to capturing and storing individual-level data electronically. All visited facilities reported having experienced periodic power outages, and only larger facilities reported having backup generators.

#### Uniquely Identifying and Linking Records

Adults (aged ≥18 years) are provided a unique national civil identification number. Although these numbers are currently not collected as part of routine health care, registers have recently been modified by the Ministry of Health to facilitate collection. Nationally, patient names (first, middle, and last names) and age are collected as identifiers in HTCs for all patients. All patients in care are assigned a unique number that is included in the PMS, regional LIMS, and EMR data warehouse. Although this number is unique within the country, it is often not transferred from one facility to the next, resulting in patients being assigned an additional number when obtaining care at a subsequent site. Names are not recorded at six of the seven regional laboratories, nor are they recorded at the EMR data warehouse. The lack of identifiers limits the accuracy of data matching and de-duplication processes.

#### Capturing Key Events From Multiple Data Sources

A high number of variables necessary for CBS are available in Kenya. The national PMS can be used to identify the number of patients in care, on ART, retained on ART, and achieving viral suppression, although many of the facilities use paper-based systems. The EMR data warehouse obtains the same variables as the PMS but is limited to facilities with an EMR. Interoperability between EMRs is currently not available as the systems have different software developers and are not designed to be networked.

Viral load testing is standard for ART patients and is performed at centralized laboratories using a LIMS, with results reported to care and treatment facilities. However, the time from obtaining the specimen to the availability of the result varies and can exceed the target of two weeks. In one laboratory, as a result of reagent stock outs, this time period exceeded six months.

A notable high-quality system is the Eastern Deanery AIDS Relief Program (EDARP), which operates fourteen health facilities. They have developed a fully integrated, networked, and paperless EMR system, which includes a LIMS and pharmacy system. Patients are entered into the EMR when they come to HTCs, allowing for tracking of patients from diagnosis into care. The system collects several identifiers, including the national identity number. It is a closed (self-contained) system that requires sustainable resources, and it highlights what can be achieved should resources be available.

#### Feasibility and Readiness of Implementing Case-Based Surveillance

The EMR data warehouse and LIMS systems are currently not sufficient for CBS, as names and national identifier are not included. Additionally, the data in the EMRs are not systematically compared against the paper medical records to assess accuracy of data entry. Limitations of the PMS for CBS are that for nearly all facilities, recording of data begins at entry to care, does not link to information on HIV diagnoses or vital registrations, and are often paper-based. Current demands on providers for patient management preclude the possibility of reporting patients for CBS, although HTC counselors could potentially report new diagnoses if variables are minimal.

### Commonalities and Differences across the Three Countries

Stakeholders in the three countries indicated substantial interest in, and support for, CBS to effectively monitor HIV. In all three countries, routine HIV program monitoring is conducted, registers and paper medical records are standardized, and variables collected are sufficient for CBS. There are efforts in South Africa and Kenya to establish national data warehouses with individual-level data.

Stakeholders expressed concern over resources, indicating that CBS would only be feasible and sustainable if it could be conducted without substantially increasing the workloads of health care providers and surveillance officers. Based on current reporting requirements, stakeholders felt that having sites report data through their existing electronic systems (EMRs and LIMS) would not require substantial additions to facility level staff but would require additional training and information technology support.

Implementing CBS based on current systems would result in gaps between care cascade indicators due to the absence of linkage between testing, care, and mortality data. Currently systems for testing and deaths are primarily paper-based with limited identifiers and are not routinely linked to health systems. Cascade of care data availability are presented in Table 2 [1].

Although EMRs are in place in each of the three countries, they vary in design, quality, and coverage of facilities. Unstable power sources and internet access was a problem often cited as it adversely impacted electronic and networked systems. Also often cited was the issue of poor data quality and difficulties in comprehensively collecting accurate personal identifying information. These and additional factors affecting the feasibility of CBS are presented in Table 3 [13].

**Table 2:** Data availability for care cascade indicators.

Cascade measure	Tanzania	South Africa	Kenya
People living with HIV diagnosed	Testing data are in paper-based registers but do not include names or other personal identifiers needed for de-duplication issues. The PMS^a^ includes date of diagnosis.	Testing data are in paper-based registers and include names and other personal identifiers that may be used for de-duplication issues. The PMS includes date of diagnosis.	Testing data are in paper-based registers and include names and other personal identifiers, although data are not sufficient for de-duplication issues. The PMS includes date of diagnosis.
HIV care coverage	Unable to determine unduplicated number of people diagnosed; therefore, the proportion of people linked to care cannot be determined. The PMS starts at entry to care.	The PMS does not currently include care information prior to starting ART^b^. Care services are sometimes offered separately from ART services.	Insufficient identifiers obtained in testing to unduplicate and determine proportion linked to care. The PMS starts at entry to care.
ART coverage	Only the proportion of people in care on ART can be determined.	Only the proportion of people in care on ART can be determined.	Only the proportion of people in care on ART can be determined.
ART retention	Can be determined at the facility level; currently unable to resolve duplication issues at the national level.	Can be determined through viral load tests noted in the PMS and LIMS^c^.	Can be determined at the facility level; currently unable to resolve duplication issues at the national level.
Viral suppression	Viral load testing for routine monitoring of patients on ART is being rolled out and is currently unavailable for most patients; tests conducted are noted in the PMS.	Viral load testing for routine monitoring of patients on ART is widely available. Test information is available in the PMS and LIMS, typically within 48 hours.	Viral load testing for routine monitoring of persons on ART is fairly recent; data are available in the LIMS and there is often a long lag time between tests and data entered into the PMS.
AIDS-related deaths^d^	Deaths are recorded in the PMS, although reporting is incomplete, especially cause of death. The death registry is a separate paper-based system and is not routinely linked to the PMS.	Deaths are recorded in the PMS. The death registry is a separate electronic system with limited access by health staff.	Deaths are recorded in the PMS, although reporting is incomplete, especially cause of death. The death registry is a separate paper-based system and is not routinely linked to the PMS.

^a^PMS: patient monitoring system.

^b^ART: antiretroviral therapy.

^c^LIMS: laboratory information management system.

^d^The ability to separately identify and report on AIDS-related deaths, as opposed to all-cause mortality amongst people living with HIV, was not assessed.

**Table 3:** Factors affecting the feasibility of case-based surveillance.

Factors	Tanzania	South Africa	Kenya
Data management	Currently de-duplication is performed only by clinical identifier. Although DQA^a^ policies are in place, they are not fully implemented.	Roll out of a patient health registration system with a unique identifier is in progress. The NHLS^b^ de-duplicates data utilizing an algorithm. DQA policies are in place and variably implemented for the PMS^c^.	In a recent CBS^d^ pilot, de-duplication was performed using an algorithm. The new EMR^e^ data warehouse de-duplicates data based on clinical identifier. Limited DQA is being conducted at facilities.
Policies	There are no policies for HIV reporting, data security, and confidentiality. Policies in place for data quality are often not being followed.	There are no policies that mandate HIV reporting. Policies are in place for data quality, security, and routine program data management. There is a policy impasse around access by health department to vital registration data.	Policies are in place for infectious disease reporting, but not specific to HIV. There are gaps in policies for data security, confidentiality, and purpose and utilization of EMRs, LIMS^f^, and the data warehouse.
Information technology	The majority of care facilities enter data into an electronic database; the database does not have connectivity and data are extracted on a quarterly basis and sent to the national level. The PMS database is national; therefore, interoperability is thought to be unnecessary. Although there is a pharmacy module in the CTC^g^ system, it is rarely utilized. Various LIMS exist although are not connected to the CTC. Internet connectivity limited in rural areas.	TIER.Net^h^ is a national system that limits interoperability issues. It is implemented off-line with quarterly dispatches sent centrally. The national LIMS captures the majority of laboratory tests, which are then checked for duplications. Regional laboratories all utilize the same LIMS to reduce interoperability issues. Internet connectivity is limited in rural areas. Staff shortages impact the quality of the implementation of the TIER.Net system.	Four main EMRs are operating at health facilities. The EMRs are not interoperable. Backup of the data varies between facilities. The quality of data in EMR systems has not been evaluated. A system is in the pilot phase to pull data from each type of EMR for CBS. Internet connectivity is limited in rural areas. Power outages are common; larger health facilities have a generator.

^a^DQA: data quality assurance.

^b^NHLS: National Health Laboratory System.

^c^PMS: patient monitoring system.

^d^CBS: case-based surveillance.

^e^EMR: electronic medical record.

^f^LIMS: laboratory information management system.

^g^CTC: care and treatment clinic.

^h^TIER.Net: Three Interlinked Electronic Registers.

## Discussion

### Principal Findings

The findings from the situational analyses support the development of CBS systems. However, none of the programs or PMS in their current forms can accurately monitor indicators along the care cascade to track progress toward the UNAIDS 90-90-90 targets.

The lack of a universal patient unique identification system in all three settings is the most important obstacle to the evolution of existing systems, whether electronic or paper-based, toward CBS because of its pivotal role in linking records across systems and resolving duplication in reports. A robust CBS system requires data collection systems that pertain to HIV infected persons to collect patient names (or an anonymized representation of their names) and a national identifier. A robust system must also maintain data confidentiality and security. Electronic medical records were present in all three settings, and they hold the potential for efficient and secure collection and transfer of data for CBS. However, investment in infrastructure and information technology is necessary to expand the use of EMRs to include information from HIV testing sites and laboratories.

To promote the collection and use of individual-level data from multiple sources through CBS, standard operating procedures and national policies need to be developed. These policies and procedures must address any legal requirements for disease reporting and endorse data security and confidentiality. It has been suggested that policies and procedures also engage civil society in the development of identifiers, promote performance standards for completeness, timeliness, and data quality, and take into consideration discriminative laws and policies on HIV, sex work, drug use, and same-sex relations [1,2,4,9].

Laboratory information management systems are increasingly common, and in many settings, viral load testing at regional laboratories is expanding, offering the potential for efficient reporting of these tests and results. Developing laboratory data are a key contributor to CBS goals. Countries should develop a harmonization strategy for laboratory data that includes comprehensive inclusion and sharing of available identifiers. The ability to retrieve system-wide laboratory data would support the development of a CBS system; ideally, such a system could also promote continuity of care as patients increasingly move between health facilities.

In all settings, there was evidence of fragmentation of information systems. Multiple systems, limited interoperability, and inadequate capacity present barriers to managing the complex functions of stewarding the information system and the interoperability environment. To overcome these barriers, national and subnational health ministries should be actively capacitated to implement and sustain these functions for clinical care, service management, and surveillance purposes. This should include ensuring that available data, including vital registration, be accessible to the health ministries.

### Limitations

Due to practical limitations, we conducted our assessments only in select regions within each of the three countries, and not all stakeholders, such as the private sector, were included in the assessment. As our aim was to assess whether existing data systems could facilitate the development of CBS in resource-limited countries, an evaluation of the data quality within each system, the potential to capture information on key populations, and the potential representativeness and timeliness of a future functioning CBS system was not performed. Despite these limitations, our findings indicate that with some important changes and additions to existing systems, CBS can be developed or strengthened in the three countries.

### Conclusions

Evidence from several settings has demonstrated the ability of CBS to systematically capture routinely collected health data to describe and monitor HIV epidemics for program planning and evaluation and, ultimately, disease control [14-17]. It is likely that countries in sub-Saharan Africa, in addition to the three assessed, have existing systems that may be improved and expanded to accommodate CBS implementation. Considering the characteristics that define CBS, these countries should carefully examine and evaluate their existing infrastructure, human resource capacity, HIV PMS, EMRs, and infectious disease reporting systems and develop a CBS system that, to the greatest extent possible, leverages current systems and staff. In implementing HIV CBS, systems need to be developed so that they are context appropriate, feasible, scalable, secure, and importantly, given the resource environment, sustainable.

## Abbreviations

ART: antiretroviral therapy
CBS: case-based surveillance
CTC: care and treatment clinic
EMR: electronic medical record
DHIS: District Health Information System
DQA: data quality assurance
HIV: human immunodeficiency virus
HTC: HIV testing center
LIMS: laboratory information management system
MeSH: Measurement and Surveillance of HIV Epidemics
MoH: Ministry of Health
NHLS: National Health Laboratory System
PMS: patient monitoring system
TIER.Net: Three Interlinked Electronic Registers
UNAIDS: United Nations Joint Program on AIDS
WHO: World Health Organization
